# Primary colonic melanoma: a rare entity

**DOI:** 10.1186/s12957-022-02721-z

**Published:** 2022-08-10

**Authors:** Junhua Xie, Gang Dai, Yuhao Wu

**Affiliations:** 1 Division of Gastrointestinal Surgery, Department of General Surgery, Fengdu People’s Hospital, Chongqing, China; 2 Department of Cardiothoracic Surgery, Children’s Hospital of Chongqing Medical University; Ministry of Education Key Laboratory of Child Development and Disorders; National Clinical Research Center for Child Health and Disorders; China International Science and Technology Cooperation base of Child development and Critical Disorders; Chongqing Key laboratory of Pediatrics, No.136 Zhongshan Second Road, Chongqing, 400014 China

**Keywords:** Melanoma, Colon, Surgery, Stomach

## Abstract

Gastrointestinal melanoma is usually metastatic in origin, and primary melanoma within the gastrointestinal tract is rarely reported. Colon is considered to be an extremely uncommon site for primary melanomas. Herein, we report the first case of a large primary melanoma within the transverse colon with gastric involvement. CT scan found a mass within the colon, which seemed to connect to the gastric antrum. Esophagogastroscopy showed an ulcerated lesion in the greater curvature of the stomach. Subsequent colonoscopy identified a large ulcerated lesion rendering significant stenosis of the transverse colon. Biopsy following colonoscopy indicated a diagnosis of colonic melanoma based on pathological findings, which identified submucosal malignant melanoma cells with epithelioid and spindle features. Immunohistochemical stains were positive for S-100, HMB-45, Vimentin, and Melan-A. A series of clinical and imaging examinations revealed no suspicious primary cutaneous or ocular lesions. The diagnosis of primary colonic melanoma was considered. A radical transverse colectomy with subtotal gastrectomy were conducted subsequently. Definite diagnosis of primary colonic melanoma can be established after ruling out the possibility of being a metastasis from other more common primary sites. Primary colonic melanomas are a challenge to diagnose and often need a multidisciplinary treatment approach, including surgery, BRAF-targeted therapy, and immunotherapy.

## Introduction

Melanoma within the gastrointestinal tract is usually metastatic in origin, and primary gastrointestinal melanoma is rarely reported. Primary melanoma can originate from the esophagus [1], small intestine [2], rectum, and anus [3]. However, colon is considered to be an especially uncommon site for primary melanoma, and the incidence of primary colonic melanoma is extremely low compared to that of other types of colon tumors [4]. The etiology of primary colonic melanoma has not been completely elucidated due to the embryologic absence of melanocytes in the colon. In the literature, there are sparse case reports of primary colonic melanomas which generally appear as isolated polyps or ulcerated lesions [5–7]. However, to the best of our knowledge, a large primary colonic melanoma which invades the neighboring organs has not been reported yet. Herein, we report a large primary melanoma within the transverse colon invading the stomach.

## Case presentation

**Fig. 1:**
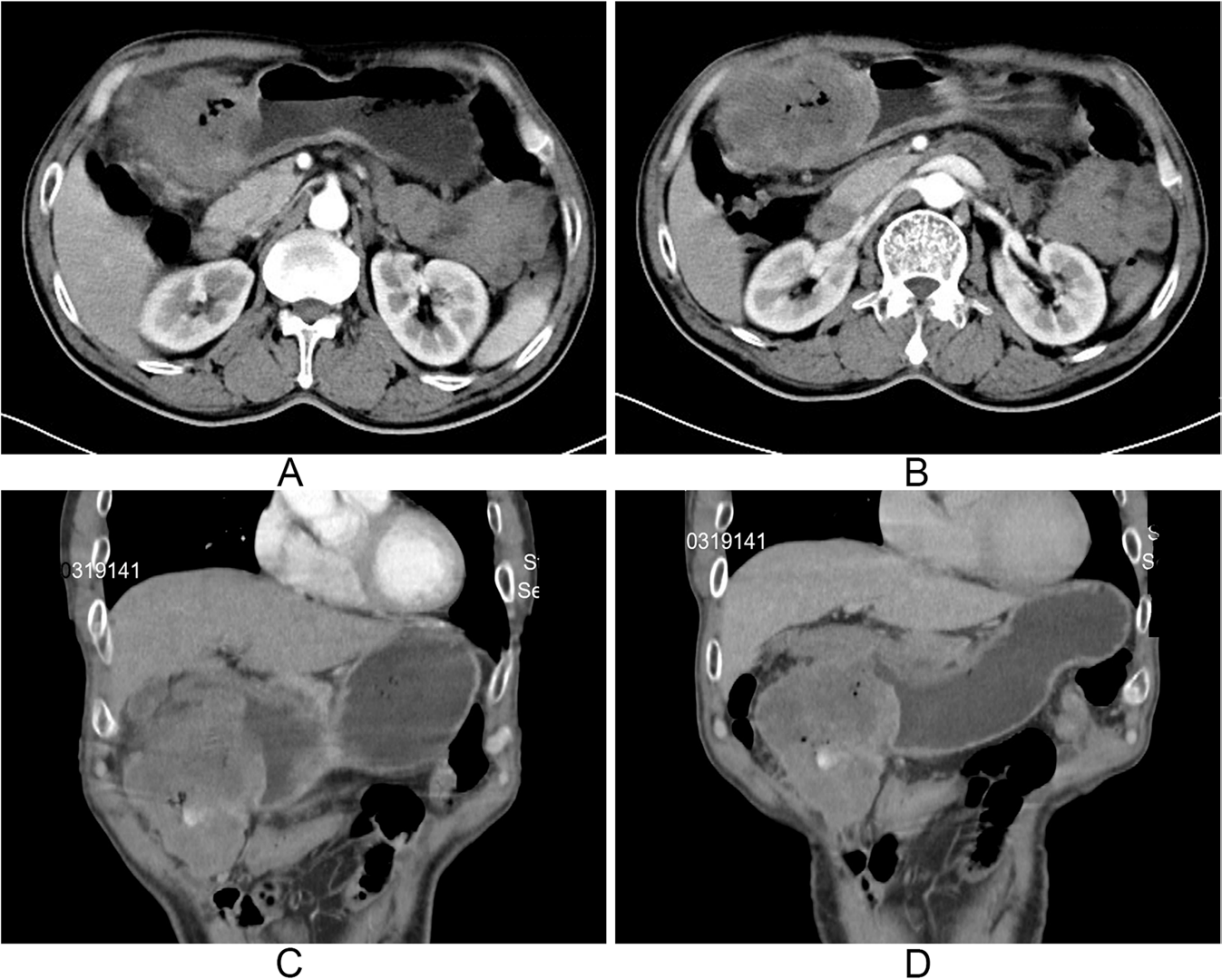
Enhanced abdominal CT scan indicated a abdominal mass with mild peripheral enhancement. The mass seemed to connect to the gastric antrum. **A**, **B** The cross-sectional images and **C**, **D** the sagittal images

**Fig. 2:**
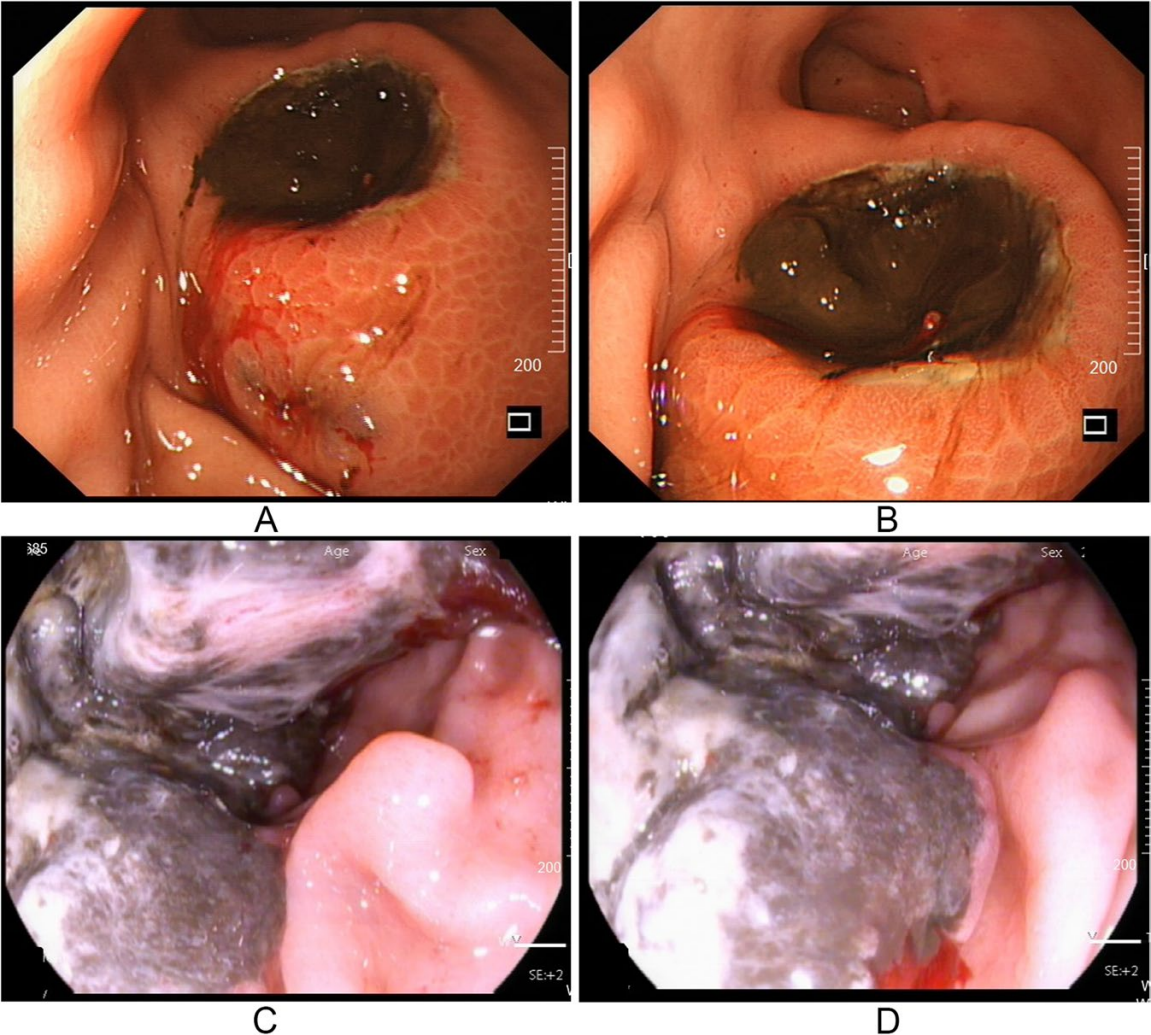
Esophagogastroscopic and colonoscopic findings. **A**, **B** Esophagogastroscopy showed an ulcerated lesion with adherent blood clot in the greater curvature of the stomach; **C**, **D** Colonoscopy showed a large ulcerated lesion with dark gray in color

A previously fit 62-year-old male presented with a 2-month history of lump in his right upper abdomen. He also complained intermittent pain in the right upper quadrant and melena. He denied any other associated diseases and family history of gastrointestinal tumors. Physical examinations revealed a movable mass in his right upper quadrant with mild tenderness, and there were no any other positive findings. Routine blood test revealed that the hemoglobin was 130 g/L. Serous CEA, CA125, and CA19-9 were negative. Abdominal ultrasonography indicated a large solid mass with sufficient blood supply. Enhanced abdominal computed tomography (CT) also found a mass within the colon, which seemed to connect to the gastric antrum (Fig. 1). Esophagogastroscopy showed an ulcerated lesion with adherent blood clot in the greater curvature of the stomach (Fig. 2A, B). Subsequent colonoscopy identified a large ulcerated lesion rendering significant stenosis of the transverse colon (Fig. 2C, D).

**Fig. 3:**
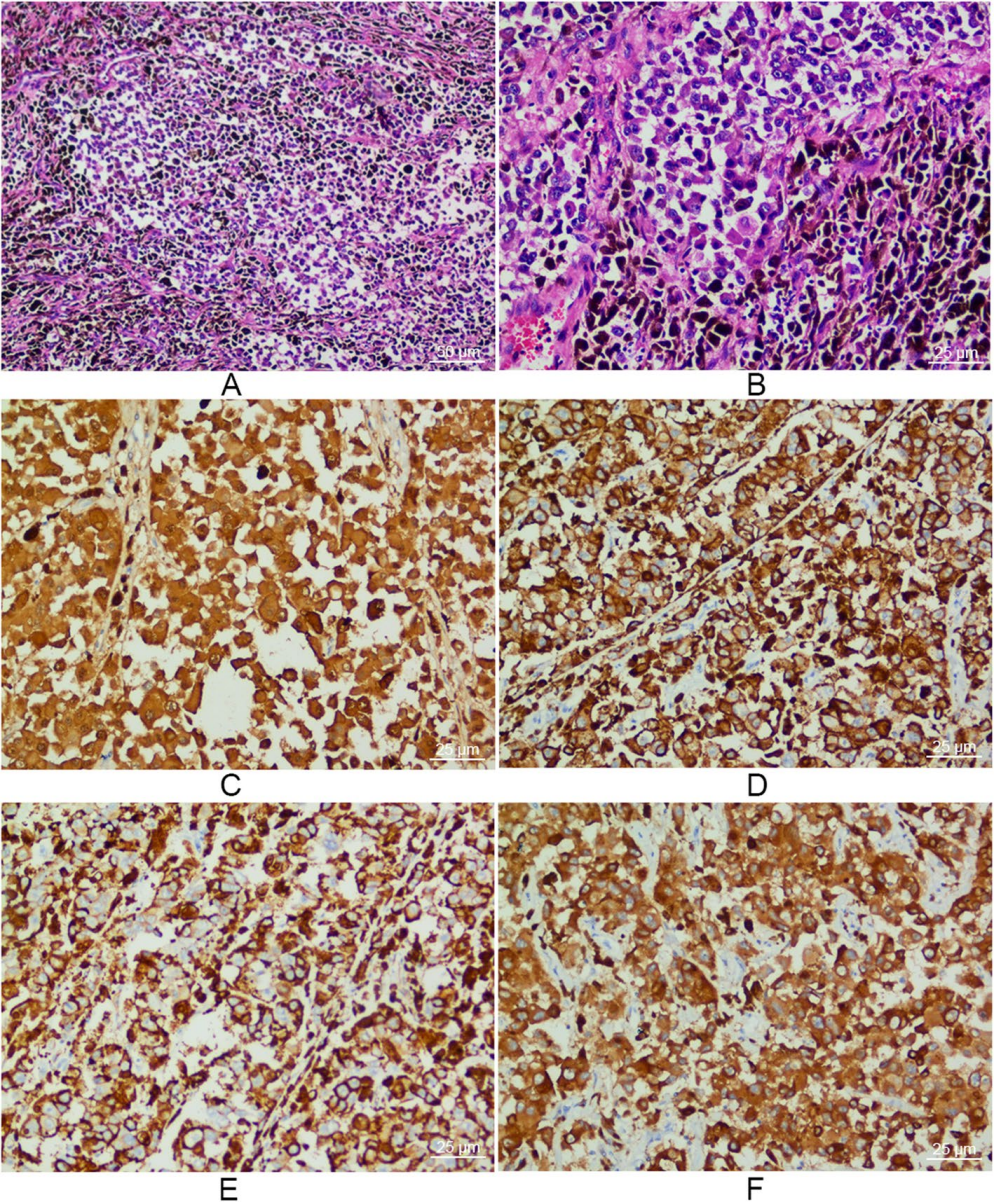
Hematoxylin-eosin (H&E) and immunohistochemical stains of resected specimens. **A**, **B** H&E stains showed melanoma cells with epithelioid and spindle morphology. Immunohistochemical stains showed positive for S-100 (**C**), HMB-45 (**D**), Vimentin (**E**), and Melan-A (**F**). Scale bar=50 or 25μm

Biopsy following colonoscopy indicated a diagnosis of colonic melanoma based on pathological findings, which identified submucosal malignant melanoma cells with epithelioid and spindle features. Immunohistochemical stains were positive for S-100, HMB-45, Vimentin, and Melan-A (Fig. 3). To exclude metastatic lesions from primary cutaneous or ocular melanoma, a thorough dermatological and ophthalmic examination was performed, but there were no suspicious skin or ocular lesions. Subsequent thoracic and cranial CT scan did not identify any metastatic lesions either. Therefore, the diagnosis of primary colonic melanoma was considered.

Subsequently, a radical transverse colectomy and subtotal gastrectomy were conducted. The gross size of tumor was 16×10 cm. 21 out of 23 pericolic lymph nodes were positive for metastasis. The patient was negative for mutations of the *c-kit* gene and V600 mutation of the *BRAF* gene. This patient refused any adjuvant immunotherapy, and he was discharged 2 weeks after surgery. Regular out-patient follow-up was required, and there has been no recurrence during the follow-up for one year so far.

## Discussion

Although melanoma most commonly arises in the skin, primary melanoma can also originate from the gastrointestinal tract. Pathogenesis of primary colonic melanoma mainly includes tumor regression and ectodermal differentiation theories [8]. Currently, evidenced-based guidelines are lacking when diagnosing primary melanoma in sites on which it rarely arises. Given that most gastrointestinal melanomas are metastatic in origin, comprehensive physical examinations and imaging studies are required. It is imperative to exclude metastatic lesions from primary cutaneous or ocular melanoma. Therefore, when there is no history of previous melanoma, a whole-body dermatological and ophthalmic examination is crucial [3]. Consistent with the criteria reported in the literature, the diagnosis of primary colonic melanoma in this case is determined based on lack of previous cutaneous melanomas, lack of other metastatic presentations, and in situ change of the gastrointestinal epithelium. To our knowledge, our case is the first reported large primary colonic melanoma invading the stomach. We speculate that the causes of gastric involvement are due to the rapid growth of primary colonic melanoma and local invasion. Although chemotherapy is suggested due to metastasis of pericolic lymph nodes, this patient declines this recommendation.

The 5-year survival rate is 33% in patients with metastatic colonic melanomas, but the prognosis of the primary colonic melanomas is comparatively better [8]. Since primary colonic melanomas are rarely reported, the risk factors for long-term survival cannot be determined based on current literature.

In conclusion, given that most colonic melanomas appear as isolated polyps or ulcerated lesions in the literature, our case is the first reported large primary colonic melanoma invading the stomach. Histopathological studies and immunohistochemical stains are essential in the diagnosis. Definite diagnosis of primary colonic melanoma can be established after ruling out the possibility of being a metastasis from other more common primary sites.

## Data Availability

Not applicable.
